# The Role of Tenascin-C in Neuroinflammation and Neuroplasticity

**DOI:** 10.3390/ijms262010174

**Published:** 2025-10-19

**Authors:** Ya-Li Jin, Shi-Wen Bao, Meng-Xuan Huang, Yong-Jing Gao, Huan-Jun Lu, Xiao-Bo Wu

**Affiliations:** Institute of Pain Medicine and Special Environmental Medicine, Coinnovation Center of Neuroregeneration, Nantong University, 9 Seyuan Road, Nantong 226019, China

**Keywords:** Tenascin-C, synaptic plasticity, immunoregulation, neuroinflammation

## Abstract

Tenascin-C (TNC) is a complex extracellular matrix (ECM) protein that plays a critical role in regulating cellular adhesion, motility, proliferation, and inflammation through its interaction with Toll-like receptor 4 (TLR4) and other receptors. The upregulation of TNC is associated with inflammatory responses, autoimmune disorders, and neoplastic conditions during both physiological and pathological tissue remodeling. In the central nervous system (CNS), TNC contributes to neuroinflammatory processes by modulating the function of immune cells and the secretion of pro-inflammatory mediators, thereby playing a pivotal role in the initiation and progression of neuroinflammatory diseases. TNC is expressed in astrocytes, neural progenitor cells, and various neuronal populations within both developing and mature CNS regions. It regulates neuronal migration and axonal guidance during neurogenesis, facilitating synaptic plasticity and CNS regeneration. Furthermore, TNC enhances neuroplasticity through interactions with receptor families, such as integrins, to establish the molecular connections necessary for cell communication and signal transduction. This review investigates the mechanistic properties of TNC, focusing on its spatiotemporal expression, molecular interactions with receptors, and its role in neurological disorders, in addition to its modulatory capacity in neuroplastic processes. Additionally, this review delves into recent research advancements with respect to neuroinflammation involving TNC, along with therapeutic strategies targeting TNC.

## 1. Introduction

Tenascins represent a class of extracellular matrix (ECM) glycoproteins that are crucial for tissue morphogenesis, expansion, and regeneration in vertebrate organisms. They are known for their ability to interact with a variety of ECM components and cellular elements, thereby influencing cellular adhesion, motility, and differentiation [1]. The tenascin gene family in vertebrates encodes four structurally related proteins: Tenascin-C (TNC), Tenascin-X, Tenascin-R, and Tenascin-W [2]. As the first member identified in the tenascin protein family, TNC plays a critical role in regulating cellular proliferation, signal transduction, phenotypic differentiation, synaptic function, and inflammatory processes [3,4,5,6]. As a secreted hexameric ECM glycoprotein, its multidomain architecture enables it to engage in complex interactions with diverse transmembrane receptor families [5]. It promotes cell growth, migration and angiogenesis through inflammatory mediation, brain lesion formation, and the activation of multiple intracellular signaling pathways [7,8,9]. Furthermore, it serves as a critical modulator in oncogenesis (particularly glioblastoma), vasculopathies, neurodegeneration, and immune dysregulation syndromes [10,11].

In physiological conditions, TNC is crucial for embryonic development and tissue homeostasis, whereas its expression in pathological states is linked to an increase in various autoimmune diseases and cancer [12]. The role of TNC within the nervous system is particularly complex. Throughout the process of neural development, TNC is abundantly expressed in neural stem and progenitor cells, orchestrating the migration of neurons and axons, and facilitating the formation of synapses and the establishment of neural networks [13,14]. During neuroinflammation, TNC upregulation triggers the stimulation of microglia and astrocytes, resulting in the secretion of inflammatory mediators [15]. This intensifies the neuroinflammatory cascade, precipitating neuronal impairments and neurodegenerative transformations. Additionally, TNC exerts a dual role in neural plasticity. Moderate levels of TNC expression facilitate the growth of neurons, the formation of synapses, and their subsequent remolding, thereby enhancing the adaptability of the nervous system [16]. Conversely, excessive TNC expression can result in synaptic dysfunctions and neuronal mortality [17]. This dual role in both neuroinflammation and neural plasticity renders TNC a pivotal target for researching neurological disorders. In recent years, as investigations into TNC’s functional significance in neural circuitry have advanced, its interplay in neuroinflammation and neural plasticity has garnered increasing attention. Moderate inflammatory responses can promote neuronal growth, synaptogenesis and neural circuit reorganization via the secretion of multiple neurotrophic factors including brain-derived neurotrophic factor (BDNF), ultimately improving neural network plasticity [18]. However, prolonged or dysregulated neuroinflammation consistently contributes to the advancement of neurodegeneration. This prolonged inflammatory state precipitates neurotoxic effects, thereby impairing neuronal function and precipitating synaptic loss and neuronal mortality [19].

TNC can modulate the balance between inflammation and neural plasticity, consequently affecting the physiological operations of neural networks and the pathogenic development of neurological disorders. Thus, elucidating the mechanisms through which TNC influences neuroinflammation and neural plasticity is important for unraveling the pathogenesis of neurological disorders and for developing novel therapeutic approaches. The critical review consolidates existing evidence on TNC’s functional roles in neuroimmunological regulation and synaptic remodeling, explores its specific mechanisms in the interaction between neuroinflammation and neural plasticity, and discusses its implications for CNS pathologies.

## 2. Neuroinflammation and Neuroplasticity

Neuroinflammation is a complex immunological response elicited by the central nervous system (CNS) in response to diverse endogenous stimuli (such as protein deposition and metabolic disturbances) or exogenous triggers (including infections and exposure to toxins) [20]. In contrast to systemic inflammation which impacts the entire organism, neuroinflammatory processes are anatomically restricted to the CNS, playing pivotal roles in maintaining neural homeostasis. Persistent or dysregulated neuroinflammation exerts deleterious consequences on neuronal physiology and cytoarchitecture, driving the development of multiple neurological pathologies. Neuroinflammatory processes involve the mobilization of heterogeneous immune cells particularly microglia and astrocytes that, when activated, release an array of intercellular signaling molecules comprising cytokines, chemokines, and related pro-inflammatory substances. Concurrently, corresponding signaling pathways are initiated, culminating in neuronal function alterations and the modulation of synaptic plasticity. Such alterations may precipitate neuronal hyperexcitability and apoptosis, thereby exerting an impact on the normal functioning of the CNS [21,22,23].

Neuroinflammation plays a pivotal role in a multitude of neurological disorders, including Alzheimer’s disease (AD), Parkinson’s disease (PD), epilepsy, and depression [20,21,24]. Neuroinflammation is a key feature of AD. It is believed to contribute to the progression of disorders by promoting the formation of amyloid plaques and neurofibrillary tangles, which are hallmark pathologies of AD [25]. Chronic inflammation can exacerbate the degradation of neurons, increase oxidative stress, and facilitate immune cell transmigration across the blood–brain barrier (BBB), potentially exacerbating neuroparenchymal injury [26,27,28]. In PD, neuroinflammatory processes correlate with the gradual degeneration of nigral dopaminergic neurons [29]. The immune-mediated reaction precipitates the secretion of cytotoxic agents that compromise cellular integrity [30]. In addition, inflammatory cascades are capable of perturbing electrophysiological homeostasis, increasing ictal predisposition, and facilitating epileptiform activity generation via inflammatory mediator production [23]. Chronic low-grade inflammation has been documented in the cerebral tissue of depressed patient, and contributes to negative impact on mood and cognitive function [31,32].

Neuroplasticity generally refers to the capacity of neurons and their networks to modify their connectivity and behavioral patterns following internal and external stimuli [33]. During the developmental process, neuroplasticity manifests through various facets, including neurogenesis and the regulation of neuronal quantity, the migration of neurons, alterations in synaptic strength and the connectivity of neural networks, alongside the emergence of novel synaptic contacts [34,35]. Neuroplasticity not only aids in adapting to the environment and acquiring novel information but also plays a pivotal role in the recovery from neurological diseases and injuries [36]. Furthermore, in conditions such as chronic pain, depression, and neurodegenerative diseases, neuroplasticity may demonstrate maladaptive modifications [37]. Research suggests that TNC is capable of modulating synaptic efficacy within hippocampal circuits in vitro. The fibronectin type III repeats 6–8 domain exhibits selective binding affinity for the perikarya of hippocampal pyramidal and granule neurons in *C57BL/6J* mice, while excluding other neuronal types, thereby modulating hippocampal-dependent contextual memory [38]. Furthermore, as a component of the ECM, TNC affects the establishment of long-term potentiation (LTP) and long-term depression (LTD) by modulating synaptic Ca^2+^ elevation, thereby controlling synaptic transmission and plasticity in the hippocampus [39]. TNC can also bind to the somata of hippocampal neurons, thereby influencing synaptic transmission processes that are integral to learning and memory. Its absence results in diminished functional plasticity in the cortex of mice following whisker trimming [40,41].

The effect of neuroinflammation on neuroplasticity is a double-edged sword (as observed in Table 1). On the one hand, neuroinflammation can promote neuroplasticity in some cases [42]. A moderate inflammatory response can enhance the adaptability of the nervous system by releasing various cytokines, including BDNF, to stimulate neurite outgrowth, the formation of synapses and their remodeling [43,44,45]. For example, during recovery from traumatic brain injury (TBI), moderate neuroinflammation can promote the recovery process by activating the regenerative and remodeling mechanisms of neurons [46]. In addition, certain studies have shown that neuroinflammation may enhance communication between neurons, thus providing beneficial neuroprotection in certain pathological conditions [47]. However, chronic or excessive neuroinflammation is often associated with the progression of neurodegenerative diseases, such as AD and PD [48]. This prolonged inflammatory state results in neurotoxic effects that impair neuronal function and induce synaptic loss and neuronal death. For instance, in AD, the continuous activation of microglia and the overproduction of cytokines may result in synaptic dysfunction and neurodegeneration [49,50].

Given its ability to modulate the balance between inflammation and neural plasticity, TNC is likely to exert influence over both processes. It may regulate inflammation levels by promoting neurite outgrowth and synapse formation, which are associated with neural plasticity during moderate inflammation, thereby facilitating injury repair. Conversely, TNC may inhibit chronic or excessive inflammation to mitigate neuronal toxicity, thus preserving normal neural plasticity.

## 3. The Structure of TNC and Its Expression Distribution Within the CNS

The TNC protein, characterized by its hexameric assembly of six disulfide-bonded monomers, constitutes a high molecular weight subunit ranging from 220 to 400 kilodaltons [5]. This subunit exhibits a modular architecture comprising four characteristic regions: an N-terminal trimerization domain, epidermal growth factor-like repeats, fibronectin type III repeats (FNIII repeats), and a C-terminal fibrinogen-like globular domain [68]. FNIII repeats are pivotal in mediating interactions with integrin family members, notably αvβ3; αvβ5; and integrins bearing β1 subunits, such as α8β1 and α9β1 [69,70]. These repeats also engage with diverse growth factors, including fibroblast growth factor (FGF), platelet-derived growth factor (PDGF), and transforming growth factor-β (TGF-β) [71]; furthermore, they establish molecular interactions with other ECM components, including fibronectin, perlecan, and laminin [72], and may engage with plasma membrane receptors, including TLR4 [73]; FNIII repeats also interact with proteoglycans, including heparin sulfate proteoglycans [71]. The concerted action of these structural domains in the ECM facilitates diverse physiological processes, encompassing cellular attachment and motility, signaling, tissue repair and regeneration, tumor progression and metastasis, and immunoregulation [74,75]. For instance, the FNIII repeats engage with TLR4 to activate the myeloid differentiation factor 88/nuclear factor-κB (MyD88/NF-κB) signaling pathway, which may promote neuroinflammation and interact with growth factors such as BDNF and TGF-β to modulate neuroplasticity, while the fibrinogen-like domain contributes to ECM cross-linking with fibronectin and perlecan. The multifunctionality of TNC positions it as a promising target for investigating and treating a spectrum of diseases, including neurological, cardiovascular, fibrotic, and oncological conditions [76,77,78]. However, the exact relationship between TNC’s structure and its function still requires further research.

The TNC protein exhibits distinct patterns of expression across developmental stages and adulthood. In initial ontogenetic stages, TNC exhibits ubiquitous expression patterns within the CNS and peripheral tissues. However, the expression diminishes significantly as individuals transition into adulthood [79]. This dynamic expression is regulated by alternative splicing at the pre-mRNA level and protein modifications, with pathological upregulation observed in conditions such as inflammation and cancer [80]. During embryonic and neonatal stages, TNC displays robust expression in the neurogenic zones and subventricular zone of the brain, harboring a high concentration of neural stem cells and their progenitors. These regions serve as pivotal sites for the vigorous self-renewal and differentiation of neurogenic stem cells [61,81]. Furthermore, throughout spinal cord morphogenesis, TNC contributes to the formation of the anteroposterior axis and influences the differentiation of spinal ancestral cells [82]. In the course of brain development in both embryonic and postnatal mice, TNC exhibits a comparable spatiotemporal pattern of expression in multipotent neuronal areas and specialized CNS subregions, particularly the retinal and cortical domains [83].

Furthermore, the persistent expression of TNC within adult neural stem cell populations implies its essential function in stem cell pool maintenance and the multipotent progenitor maturation of these cells [10]. TNC demonstrates temporally restricted expression patterns within the mesenchymal compartments of developing glandular structures (including mammary tissue, odontogenic organs, and renal systems) while concurrently exhibiting embryonic expression in chondrogenic tissues, connective tissue attachments, musculoskeletal interfaces, osseous coverings, myofibroblastic layers, and chondral sheaths [16]. TNC demonstrates selective tissue distribution in physiologically normal adult organisms, being predominantly localized to hematopoietic (bone marrow and thymus) and secondary lymphoid organs (spleen and lymph nodes), wherein it may facilitate immune cell expansion, lineage specification, and functional maturation [7].

TNC expression is markedly upregulated in various pathological conditions in adults [76]. Notably, this upregulation is particularly pronounced during inflammatory processes, including chronic inflammation related to rheumatoid arthritis [12,84]. Upregulation also occurs during tumorigenesis, as in glioma [84,85]. In inflammatory heart diseases such as myocardial infarction and viral myocarditis, TNC exacerbates adverse ventricular remodeling by amplifying inflammation and fibrosis, with its serum levels serving as a prognostic biomarker for poor clinical outcomes [2]. TNC transcriptional regulation responds to repetitive tensile stress, with its upregulated expression in mechanically challenged tissues, including vascular smooth muscle fascicles, ligamentous restraints, and dense regular connective tissues [86].

## 4. TNC Plays an Important Role in the Development of CNS Diseases

Upon injury to the CNS, TNC is re-expressed, and its expression levels are intimately associated with numerous pathological cascades, encompassing cellular adhesion, migration, and the growth of neurites [1]. In the event of CNS injury, such as intracerebral hemorrhage (ICH), TNC exacerbates secondary brain damage by inducing apoptotic neuronal demise and neuroinflammation. The molecular pathogenesis principally comprises TNC activating signaling pathways associated with platelet-derived growth factor receptors, thereby facilitating neuronal necrosis and inflammation and further intensifying injuries following ICH [17]. TNC also plays a pivotal role in certain repair processes following neural injury. For instance, after sciatic nerve transection, TNC expression is upregulated at the injury proximal nerve. Through the synergistic actions of Schwann cells and neurofibroblasts, TNC can facilitate the growth and repair of nerve fibers [56]. Additionally, in post-ischemia brain tissue, TNC deficiency impaired microglial migration with respect to astrocytes, prolonging interactions and increasing astrocytic responses, via ICAM1 signaling [87].

Emerging evidence positions exosomes and other extracellular vehicles (EVs) as the dominant conduit for secreting and intercellularly shuttling TNC. Across a spectrum of cell types from fibroblasts to cancer cells, EV biogenesis, particularly exosome release, is indispensable for the correct deposition of TNC into the extracellular milieu [78]. When this pathway is disrupted whether by pharmacologically inhibiting neutral sphingomyelinase 2 or genetically deleting Caveolin-1 (*Cav1*), TNC is retained intracellularly, and the extracellular TNC-fiber assembly collapses. The sorting of TNC into specific EV subsets is critically dependent on *Cav1*, which modulates cholesterol levels within endosomal compartments. Cav1 orchestrates the selective loading of TNC onto specific EV subsets by governing cholesterol homeostasis within endosomal compartments. Once released, TNC-laden EVs can nucleate fresh extracellular-matrix foci at their origin or travel systemically to seed TNC-rich microenvironments, most notably pre-metastatic niches in distant organs. Importantly, EV-associated TNC remains bioactive, reprogramming recipient cells by driving proliferation, invasiveness, epithelial-to-mesenchymal transition (often via WNT/β-catenin signaling) [88], and immune evasion (e.g., suppressing T-cell activation through integrin engagement). Thus, EV-mediated transport emerges as a principal—perhaps even primary—mechanism of TNC trafficking and function, underscoring its contributions to tumor progression [89], cardiovascular pathology, and neuroinflammation. Circulating TNC^+^ EVs not only hold promise as clinical biomarkers but, when purified, could also sharpen the detection of tumor-specific mutations in liquid biopsies [90].

TNC, as an inducible, non-structural and secreted multifunctional protein is highly associated with disease progression and can be detected in the blood or cerebrospinal fluid (CSF), which likely renders it a clinical marker. Subarachnoid hemorrhage (SAH) from aneurysm rupture is devastating, with early brain injury (EBI) determining outcomes and causing delayed cerebral ischemia (DCI). EBI involves global edema (cytotoxic/vasogenic), and it is highly linked to TNC. Its knockout reduces EBI by inhibiting the post-SAH activation of mitogen-activated protein kinase (MAPK) and NF-κB signaling. Moreover, severe SAH increases TNC in CSF and blood, which serves as a surrogate marker for EBI and a predictor of DCI [91,92]. In TBI patients, elevated serum levels of TNC are strongly associated with the severity of trauma and clinical outcomes [59]. Future research should validate TNC’s diagnostic or prognostic utility across these conditions and explore TNC-targeted therapies to improve patient outcomes.

## 5. The Role and Mechanisms of TNC in Neuroinflammation

### 5.1. Expression of TNC in Neuroinflammatory Conditions

In the context of CNS infections and injuries, the alterations in the TNC transcriptional patterns and their pathological implications have garnered increasing attention. Previous studies indicates that in infectious diseases such as bacterial meningitis, viral meningitis, viral encephalitis, and Lyme neuroborreliosis, there is a marked elevation of TNC levels in cerebrospinal fluid, suggesting a potential role for this protein as an inflammatory mediator in the modulation of neuroinflammatory responses and tissue damage [93]. Following acute neurological insults, including cerebral ischemia, craniocerebral trauma, and subarachnoid bleeding, TNC demonstrates marked transcriptional activation. In stroke models, the suppression of TNC expression has been shown to diminish neuronal cell death and neuroinflammation, thereby alleviating brain damage, indicating that TNC may exacerbate pathological progression by promoting inflammatory responses [51,55]. Similarly, increased concentrations of TNC in the CSF of TBI patients are associated with adverse outcomes, supporting its potential role as a biomarker of trauma severity [59,60]. Post-SAH, TNC participates not only in the initial phases of brain injury but additionally demonstrates robust association with delayed cerebral ischemia, further emphasizing its critical function in neuroinflammation and cerebrovascular dysfunction [57,94]. TNC participates in cardiovascular remodeling and fibrosis through TLR4-induced epithelial–mesenchymal transition. Studies have shown that TNC drives pathogenic valvular architectural alterations in two large animal models of post-ischemic mitral valve incompetence (a common complication of myocardial infarction) [95]. Furthermore, Xie et al. reported that TNC is a molecule involved in enhancing the chronic neuroinflammatory response in AD [62]. A lack of TNC enhances the detrimental effects of the overexpression of the mutated amyloid precursor protein, increases the number of microglial cells in the hippocampus and adjacent brain areas, convert the neuroinflammatory response from a pro-inflammatory to an anti-inflammatory state, attenuates cerebral β-amyloid deposition, and protects the neurons of AD mice, resulting in the beneficial modulation of the pathogenesis of AD [64].

### 5.2. Mechanisms of TNC in Modulating Neuroinflammation

**Figure 1:**
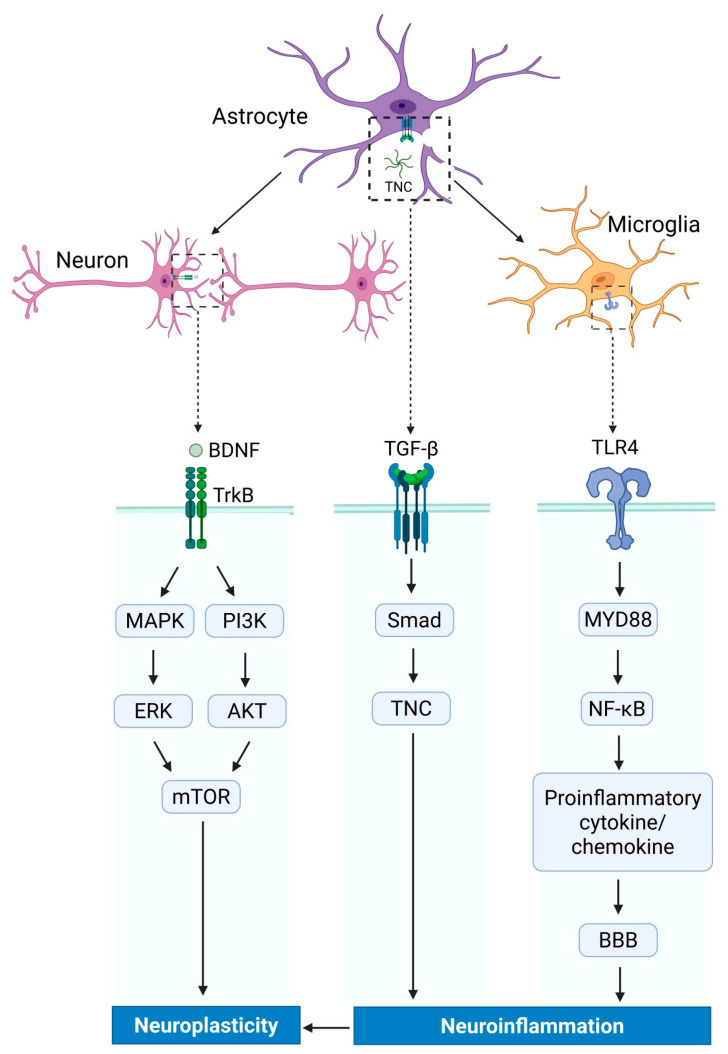
TNC orchestrates neuron–glia neuroimmune signaling in three steps. Astrocyte-derived TNC engages the microglial TLR4 → MyD88/NF-κB → secretion of cytokines and chemokines which compromises the BBB. In neurons, TNC modulates BDNF binding to TrkB, triggering MAPK/ERK/mTOR and PI3K/AKT/mTOR cascades that underlie synaptic plasticity. TNC also participates in a TGF-β–driven feedback loop: TGF-β binds its receptors on glia, activates Smad-dependent transcription, and upregulates TNC gene expression, thereby sustaining ECM–immune crosstalk. Neuroinflammation caused by activated glial cells may further bidirectionally regulate neural plasticity. TGF-β attaches to cell-surface receptors and initiates the Smad protein, which translocated into the nucleus to modulate the transcription of the TNC gene. TLR4: Toll-like receptor 4; TGF-β: transforming growth factor (TGF)-β; MAPK: mitogen-activated protein kinase; BDNF: brain-derived neurotrophic factor; PI3K: phosphatidylinositol 3-kinase; ERK: extracellular regulated protein kinases; AKT: akt serine/threonine kinase; mTOR: mechanistic target of rapamycin kinase; Smad: suppressor of mother against decapentaplegic; TNC: tenascin-C; MYD88: MYD88 innate immune signal transduction adaptor; NF-κB: nuclear factor-kappa B; TrkB: tropomyosin receptor kinase B; BBB: blood–brain barrier.

TNC participates in the regulation of neuroinflammatory responses through a variety of mechanisms (as shown in Figure 1). It interacts with membrane-bound receptors in immune cells, regulating their activation, migration, and functionality [96]. At the molecular level, TNC interacts with and modulates the function of pro-inflammatory mediators (including TNF-α and IL-1β) and immunosuppressive cytokines (including IL-10), exhibiting a bidirectional immunomodulatory effect. These cytokines can induce neuronal damage, thereby influencing the inflammatory state within the nervous system [15,97,98]. It is noteworthy that TNC, by binding to a variety of chemokines (including CCL2, CCL26, CXCL8, CXCL10, and CXCL12), regulates the chemotactic movement of immunocytes, thereby affecting the dissemination of inflammatory reactions and the infiltration of tissues [99]. Within the CNS, TNC promotes the activation of microglia and the secretion of pro-inflammatory mediators via the TLR4/MyD88/NF-κB signaling cascade, intensifying neuroinflammatory responses and causing additional brain injury under pathological circumstances like SAH and ICH [17,52,76]. In CNS injury models, TNC has been recognized as a modulator of microglial and astrocytic activation, thereby modulating the production and action of IL-1β [100]. The role of TNC in immune response and glial activation, particularly its interaction with TLR4 and its impact on microglia and astrocytes in neurodegenerative diseases, such as AD and multiple sclerosis, has been well elucidated in previous studies [15,99]. This study further explores the mechanism by which TNC participates in neuroinflammation and neuroplasticity through other signaling pathways. TNC can affect the inflammatory microenvironment of the CNS by regulating the integrity of the BBB and the activation status of glial cells. In the mouse experimental model of SAH, TNC deficiency has been shown to alleviate brain edema and BBB disruption [53]. TNF-α is a pro-inflammatory cytokine known to impair BBB integrity, and TNC may exacerbate the BBB disruption caused by TNF-α [54,101]. The expression of TNC is closely associated with various inflammatory signaling pathways and may be involved in immune regulation during pathological processes such as viral infections.

## 6. The Role of TNC in Neuroplasticity

### 6.1. Effects of TNC on Synapse Formation and Function

TNC affects synapse formation by engaging with neuronal surface receptors and altering the structural plasticity of the peri-synaptic network [102,103]. Evidence indicates that TNC can affect the formation of both inhibitory and excitatory synapse on hippocampal neurons [66]. The fibronectin type III repeats 6–8 fragment of TNC exhibits an inhibitory influence on the neurite growth of pyramidal cells and plays a regulatory role in hippocampal structural plasticity [38]. Moreover, TNC promotes neurite growth through interactions with other cell adhesion molecules. For example, the HNK-1 antigenic determinant on TNC promotes neurite elongation in hippocampal cells through its association with neurophilin-1 [104]. Beyond influencing synaptic formation, TNC also participates in the modulation of synaptic function. Studies reveal that TNC can affect neurotransmitter release and receptor function. TNC is able to function as an intrinsic agonist of TLR4, regulating chemotactic migration, phagocytic activity, and the synthesis of pro-inflammatory cytokines in microglial cells [105]. By activating TLR4, TNC indirectly impacts synaptic transmission and neuronal excitability. In the context of cerebral ischemia, TNC limits the infiltration of leukocytes, particularly T cells, into ischemic brain tissue, thereby protecting neurons and synapses from immune-mediated damage [105]. TNC modulates N-Methyl-D-Aspartate (NMDA) receptors and L-type voltage-gated Ca^2+^ channels, which are pivotal factors in initiating activity-dependent synaptic alterations [106,107]. During olfactory bulb development, TNC functions as a repulsive boundary cue, restricting the growth of olfactory sensory neuron axons, and thereby promoting axonal sorting [108]. Furthermore, studies have shown that the elimination of TNC and other ECM molecules including TNR, brevican, and neurocan, disrupts the equilibrium between excitatory and inhibitory synaptic connections [109]. The molecular mechanisms dictating TNC’s switch between pro- and anti-synaptogenic roles require further investigation, as do species-specific variations. Additionally, the manner by which TNC interacts with other ECM molecules (e.g., TNR, brevican) to maintain excitatory–inhibitory balance requires deeper exploration. Future research should prioritize interventions targeting TNC in neuropathological conditions, such as stroke and neurodegenerative diseases, to harness its neuroprotective properties. Investigating the interactions between TNC, microglia, and myelination could provide valuable insights into demyelinating diseases. Overall, TNC presents itself as a promising target for modulating synaptic plasticity, with implications for developmental neurobiology and therapeutic strategies.

### 6.2. TNC’s Involvement in Neurogenesis and Developmental Processes

TNC demonstrates pronounced expression throughout embryogenesis and has been detected in astrocytic lineages during neural development [16]. A previous study has briefly summarized TNC’s role in neuronal migration and axonal guidance during development (15), without addressing neuroplasticity. Here, current evidence suggests that astrocytic and radial glial progenitor populations serve as the principal cellular sources of TNC induction, with this glycoprotein fulfilling essential functions during neurodevelopment. It functions as an inhibitory ECM component that restricts neuronal and astrocytic outgrowth, orchestrating the following: (1) mitotic expansion and process outgrowth of astroglia precursors, (2) differentiation of neural progenitor populations, (3) clonal expansion and preservation of oligodendrocyte precursor cells, and (4) modulation of synaptic plasticity via both autocrine and paracrine signaling cascades during neurodevelopment [13,85,100,110,111,112].

During the development of the CNS, TNC is expressed in specific spatial and temporal patterns in distinct brain regions. In adults, TNC is mainly expressed in the activated neurogenic sites and regions of neural synaptic plasticity. The intra-hippocampus injection of recombinant TNC fragments encompassing fibronectin type III repeats 6–8 impedes memory maintenance and hippocampal neurogenesis in mice, which show the pivotal role of TNC in hippocampal-dependent situational memory and synaptic plasticity [112,113]. Studies should investigate the mechanistic interactions of TNC with signaling pathways, its dysregulation in neurodevelopmental disorders, and its potential as a therapeutic target. Elucidating its dynamic roles throughout the lifespan may reveal novel strategies for enhancing neuroplasticity and addressing cognitive impairments.

### 6.3. The Role of TNC in Neuronal Morphology and Neural Network Remodeling

TNC assumes a crucial function in regulating the structural remodeling of the perineuronal nets (PNNs), which represents a compact configuration of the ECM enveloping neuronal cell bodies and are essential for the adaptive modifications of the nervous system [66]. The absence of TNC can impair the quantity, intensity, and architecture of the PNN, in addition to the establishment of inhibitory and excitatory synaptic connections in PNN-associated neurons [66]. Under pathological states, including neurodegenerative disorders, post-traumatic stress disorder (PTSD), and spinal cord lesions, impaired synaptic adaptability limits the establishment of novel neural circuits, while PNN substantially restrict neuroplastic capacity [67]. Consequently, the functional contribution of ECM glycoprotein TNC within the dorsal hippocampal formation may be elucidated through examinations of the following: (1) structural modifications in PNN organization, and (2) alterations in the balance between Gamma-Aminobutyric Acid-ergic (GABAergic) and glutamatergic synaptic transmission onto these neuronal populations. In TNC^−/−^ mice, the absence of TNC resulted in a deteriorated PNN lattice ultrastructure and enhanced inhibitory innervation in the dentate gyrus, and it simultaneously augmented the quantity of PNN and inhibitory projections to the CA2 area [114]. Further research should investigate interventions targeting whether regulating TNC can reverse PNN rigidity in PTSD and neurodegeneration, aiming to restore synaptic plasticity. Understanding TNC’s interaction with GABAergic/glutamatergic systems and PNN dynamics could reveal therapeutic targets. Translational studies confirming TNC’s role in human neuroplasticity may connect preclinical findings to regenerative circuit repair strategies.

### 6.4. The Bidirectional Regulation Between ECM Remodeling and Neuronal Activity

**Figure 2:**
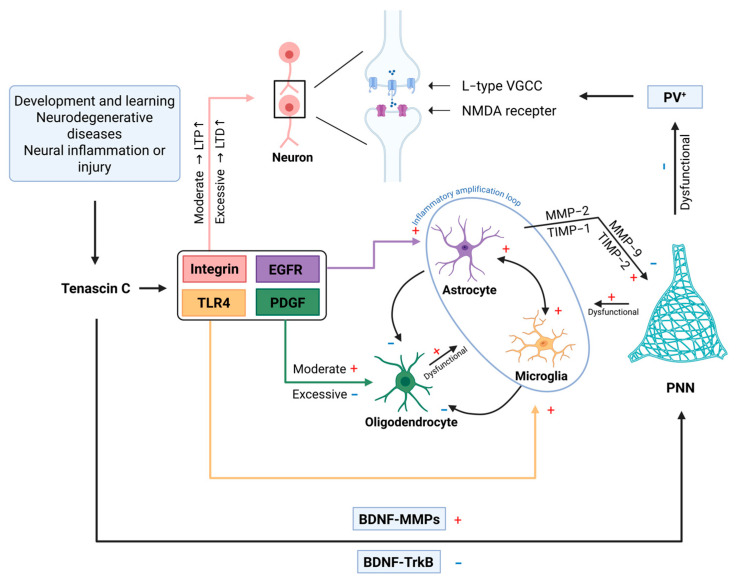
The mechanism of TNC’s effect on neural plasticity. Under various physiological (e.g., development, learning/memory) and pathological (e.g., neurodegeneration, inflammation) conditions, TNC influences synaptic plasticity through two major pathways. Direct pathway: TNC engages integrins to modulate NMDA receptor activity and L-type voltage-gated Ca^2+^ channels, thereby regulating the calcium threshold required for LTP versus LTD; Indirect pathway: TNC triggers astrocytes to secrete matrix metalloproteinases (MMP-2/9) and the tissue inhibitors of metalloproteinases (TIMP-1/2), resulting in focal degradation or the protection of perineuronal nets (PNNs). Microglia and oligodendrocytes contribute to PNN homeostasis. BDNF further bidirectionally regulates PNN integrity via TrkB-dependent or MMP-dependent mechanisms. Altered PNN composition modulates parvalbumin interneuron excitability, forming a feedback loop that ultimately determines the direction of neural network plasticity. The symbol “↑” represents the protein or related activity elevation, the symbol “+” indicates activation or potentiation, and the symbol “-” denotes suppression or functional impairment.

PNNs are special ECM aggregates rich in hyaluronic acid, aggrecan, and TNC. They can not only regulate neuronal activities but also be reversely regulated via neuronal activities (as shown in Figure 2). This two-way dialogue is crucial for plasticity and diseases. In the epilepsy model, high-frequency discharges activate the Ca^2+^/calpain-matrix metalloproteinase-9 (MMP-9) cascade, inducing an increase in the expression of ADAMTS4. This results in the degradation of the chondroitin sulfate proteoglycans (CSPGs) component of the PNN, relieving GABAergic inhibition [115]. It was found that in chronic inflammatory pain (complete Freund’s adjuvant model), the density of PNN in the somatosensory cortex (SSC) increased significantly after 3 and 7 days of persistent pain [116]. By blocking the potassium (K^+^) current of parvalbumin (PV) neurons, the power of gamma oscillations is reduced [117]; for example, the pathological remodeling of PNNs is postulated to disrupt cortical processing, potentially contributing to hyperalgesia. The chondroitin sulfate–glycosaminoglycan (CS-GAG) chains of PNNs in the arcuate nucleus (ARC) region of the hypothalamus impede insulin penetration through electrostatic repulsion. Targeted degradation of PNN can restore insulin sensitivity [118]. In addition, neuronal activity drives the remodeling of the ECM, which can also regulate neuronal activities in a reverse manner. After a spinal cord injury, TNC is deposited in the PNN, where it activates TLR4 in microglia, driving the release of interleukin-1β (IL-1β), promoting astrocytes to synthesize CSPGs, and forming an “inflammation-fibrosis” cycle [119]. The inhibition of MMP activity selectively blocks the enhancement of the non-deprived eye response after monocular deprivation (MD) without affecting the weakening of the deprived eye response, and it significantly reduces the increase in the dendritic spine density of layers II–III pyramidal neurons, suggesting that MMPs are involved in synaptic remodeling [120]. The genetic knockout of aggrecan results in the disappearance of PNN structures, and the visual cortex of adult mice regains juvenile-like ocular dominance plasticity. After the targeted degradation of PNNs, the PV neuron population shifts to a highly plastic state, with reduced synaptic constraints [121]. The spatiotemporal dynamics of PNNs (such as TNC and aggrecan) remodeling across brain regions should be investigated, and precise, context-specific interventions should be developed.

### 6.5. The Relationship Between TNC and BDNF

BDNF serves as a key neurotrophic regulator that is essential for neural system ontogeny, maintaining the survival of mature neuronal populations and stimulating de novo neuron generation [122]. Through its interaction with tropomyosin receptor kinase B (TrkB) receptors, BDNF exerts essential regulatory effects on neuronal viability, morphological remodeling, and synaptic adaptability [123]. Furthermore, BDNF facilitates axonal growth and extension, serving as a synaptic modulator [123]. The release of BDNF and the activation of TrkB are contingent upon MMP-9, which is instrumental in structural LTP [124]. This demonstrates that the remodeling of the ECM, including the regulation of TNC, may influence BDNF-mediated synaptic plasticity. TNC interacts with transmembrane receptor complexes such as integrins, activating signal transduction, which interacts with the BDNF/TrkB signaling pathway. For instance, the TNC-induced autocrine production of PDGF stimulates the excessive proliferation of glioblastoma cells [125]. TNC is involved in the regulation of MMP expression [76], and MMPs are crucial for the release of BDNF and synaptic plasticity [124]. The relationship between TNC and BDNF is multifaceted, encompassing neurogenesis, synaptic plasticity, neuroinflammation, and neurodegenerative diseases [122]. TNC influences BDNF signaling by modulating the extracellular environment, cellular signaling, and MMP activity. Further investigation into the interactions between TNC and BDNF may unveil novel therapeutic targets for neurological disorders.

## 7. The Interplay Between TNC, Neuroinflammation, and Neuroplasticity

### 7.1. Signal Pathway Involved in TNC Regulation

Neuroinflammation can modulate TNC expression through various signaling pathways, including TLR4, TGF-β, BDNF/TrkB, and MAPK pathways. TLR4 is a pattern recognition receptor and also serves as a downstream receptor for TNC, playing a pivotal role in neuroinflammation. Accumulated experimental evidence reveals a significant correlation between the activation of the TLR4 signaling pathway and the onset and progression of neuroinflammation [126]. TNC, acting as an endogenous activator of TLR4, can initiate TLR4 activation. Upon activation, TLR4 triggers downstream signaling pathways, including those reliant on MyD88 or independent of MyD88, which, in turn, activate NF-κB and MAPK, resulting in the release of pro-inflammatory cytokines (such as IL-1β and TNF-α) and chemokines and exacerbating neuroinflammatory responses [73,126]. TLR4 is a pattern recognition receptor that recognizes pathogen-associated molecular patterns such as lipopolysaccharides (LPSs), and the damage-associated molecular patterns of Gram-negative bacteria [127]. The activation of TLR4 recruits intracellular adaptor proteins MyD88 and Toll/IL-1R domain-containing adaptor-inducing IFN-β (TRIF), which, in turn, activate downstream signaling pathways [128]. In the MyD88-dependent signaling pathway, LPS binds to the LPS-binding protein; then the LPS-LBP complex interacts with CD14; and finally, CD14 presents LPSs to the TLR4-MD-2 complex in the cell membrane [129]. This pathway mainly recruits interleukin 1 receptor associated kinase 4 (IRAK4), IRAK1 and TNF receptor associated factor 6 (TRAF6) molecules through MyD88; activates NF-κB and MAPK signaling pathways; and ultimately promotes the production of pro-inflammatory cytokines such as IL-1β, IL-6 and TNF-α [127]. In the MyD88-independent pathway, TLR4 recruits TRIF through TRAM [127]. TRIF activates TNF receptor associated factor 3 (TRAF3)and TRAF6 [127]. TRAF3 activation produces type I interferons (IFN-β and IFN-α) by activating IRF3 [127]. This pathway mainly recruits molecules such as TBK1 and IKKε through TRIF, activates the IRF3/7 signaling pathway, promotes the production of type I interferons, and initiates an antiviral immune response [127,128]. However, the specific mechanism of how TNC affects the downstream signaling pathway of TLR4 is not fully understood. TNC may affect the spatial localization of TLR4 on the cell membrane, thereby affecting its binding to MyD88 or TRIF. TNC may also affect post-translational TLR4 modifications, such as phosphorylation or glycosylation, which may alter the affinity of TLR4 with different adaptor proteins. The engagement of TLR4 signaling cascades in AD pathophysiology demonstrates concurrent involvement in neuroinflammatory processes and cognitive decline [63,130]. Suppression of the TLR4 signaling cascade reduces neuropathological inflammation while restoring normative cognitive processing [63,130]. In models of TBI and ICH, TLR4 activation amplifies neuroinflammatory responses, resulting in secondary brain injury [57,58]. Simultaneously, serum TNC levels correlate with the severity and prognosis of acute ICH.

Previous studies indicated that the distinct polarization states of microglia may affect TNC-mediated neuroinflammation [15]. In this study, we further elucidate the potential mechanisms by which TNC is involved in neuroinflammation, encompassing its roles in synaptogenesis, neural network remodeling, and its influence on ECM dynamics and signaling cascades such as PI3K/Akt and MAPK/ERK. The TGF-β/Smad axis critically governs TNC production. Upon receptor activation, TGF-β triggers Smad complex formation which translocates to the nucleus to direct TNC gene transcription [4,131]. The TGF-β signaling cascade exerts biphasic control over neuroinflammatory processes. On the one hand, TGF-β can suppress inflammatory responses and promote neuroprotection [132]. On the other hand, TGF-β may also facilitate certain neuroinflammatory responses [133]. Experimental evidence demonstrates that neuroinflammation can affect the activity of the BDNF/TrkB signal transduction, thereby influencing neuronal survival, differentiation, and synaptic function [123,134]. In some cases, activation of the BDNF/TrkB signaling pathway can alleviate neuroinflammation and enhance neuroprotective effects [45,135]. Conversely, impaired BDNF/TrkB signal transduction may exacerbate neuroinflammatory responses, resulting in cognitive dysfunction [136]. TNC is an ECM protein involved in tissue remodeling and inflammation, this is potentially regulated via BDNF/TrkB signal transduction, contributing to the pathophysiology of neuroinflammation. TNC interacts with latent TGF-β complexes through its fibronectin type III repeats, facilitating the release and activation of TGF-β [137]. TNC may also modulate TGF-β-mediated signaling cascades by controlling endoglin transcriptional levels, a co-receptor for TGF-β [138]. We hypothesize that TNC may function as an effector molecule within the BDNF-TrkB signaling cascade, participating in the regulation of neuroinflammation. The MAPK cascade represents an evolutionarily conserved regulatory network that orchestrates diverse biological processes, encompassing cellular expansion, phenotypic specialization, programmed cell death, and immune responses [139]. TNC exerts influence on the MAPK signaling pathway through multiple mechanisms. It may indirectly regulate the MAPK pathway by affecting integrin signaling, thereby impacting cytoskeletal remodeling and migratory capabilities [140]. In neuroinflammation, the MAPK pathway encompasses the ERK, c-Jun N-terminal kinase (JNK), and p38 MAPK subfamilies. TNC can activate immune cells, inducing the secretion of pro-inflammatory mediators and exacerbating inflammatory cascades. For instance, TNC can activate TLR4, subsequently activating downstream MAPK, AKT, and Wnt signaling pathways, which enhances the directional migration of BMSCs [141]. In the SAH model, TNC knockdown attenuates neuroinflammatory responses and programmed cell death via the modulation of the PI3K/Akt/NF-κB signaling cascade [133]. Future research should elucidate the modulation of TLR4 signaling by TNC, including aspects such as spatial localization and phosphorylation. Investigating the interactions between TNC and the TGF-β, BDNF, and MAPK pathways is crucial. Thus, targeting TNC or its related pathways may offer therapeutic potential for alleviating neuroinflammation associated with AD, TBI, and IHC, thereby potentially restoring cognitive function.

### 7.2. The Balancing Role of TNC in Neuroinflammation and Neuroplasticity

**Figure 3:**
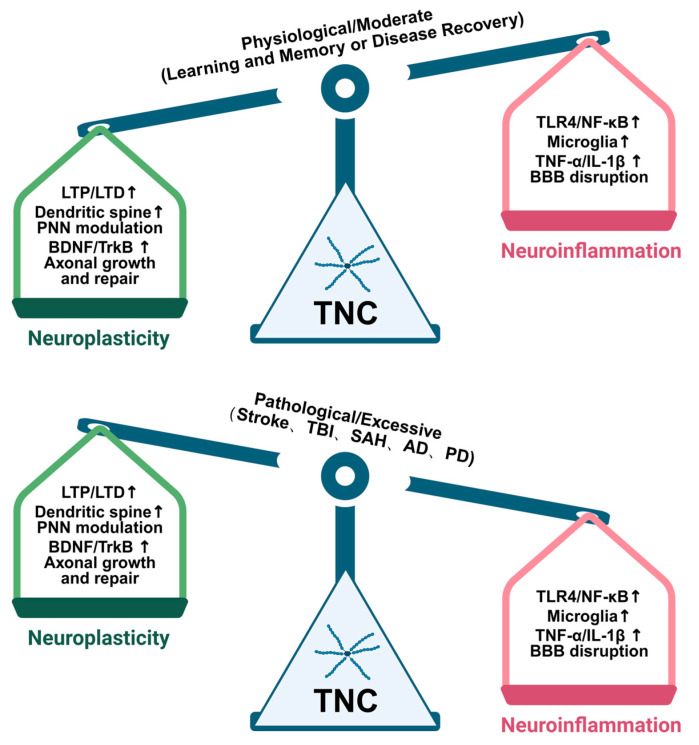
The dual and context-dependent roles of TNC in neuroinflammation and neuroplasticity. In conditions such as stroke, TBI, and SAH, TNC aggravates neuroinflammation by stimulating microglia through TLR4 signaling, resulting in the release of pro-inflammatory cytokines (e.g., TNF-α, IL-1β) and BBB disruption, which collectively worsen secondary brain injury. Moreover, in neurodegenerative diseases, such as AD and PD, TNC upregulation can accelerate disease progression by promoting amyloid-β deposition and dopaminergic neuron degeneration. On the other hand, TNC also contributes to reparative processes by supporting tissue reorganization and axonal regrowth. TNC deficiency is shown to offer neuroprotection by reducing the pathological burden and synaptic dysfunction. The diagram illustrates that TNC’s overall effect, whether harmful or helpful, is highly dependent on the specific disease environment, temporal phase (acute vs. chronic), and cellular context, underscoring its role as a key regulator at the crossroads of neuroinflammation and neural circuit adaptability. The symbol “↑” represents elevation, augmentation, or upregulation.

Inflammation alters the synaptic transmission of glutamate, thereby influencing neuronal activity and plasticity [142]. TNC exerts a profound influence on synaptic plasticity, a relationship that is inextricably linked to neuroinflammation. The absence of TNC precipitates a defect in hippocampal LTP, a form of synaptic plasticity that is contingent upon L-type voltage-gated calcium channels [107,143]. Furthermore, TNC exerts its influence on neuronal plasticity by modulating the structure and function of PNN [66]. Perineuronal nets, an ECM surrounding neurons, are indispensable for the stability and plasticity of synapses. Following brain injury, such as stroke or SAH, TNC demonstrates significant transcriptional upregulation, engaging in the processes of tissue reorganization [55,91]. The absence of TNC can mitigate neuronal apoptosis and neuroinflammation, thereby ameliorating the prognosis following SAH [57]. Neuroinflammation holds a dual-edged role in neuroplasticity; moderate levels can foster neuroplasticity, whereas dysregulated neuroinflammation accelerates the pathogenesis of neurodegeneration (as shown in Figure 3). TNC’s role in neuroinflammation is multifaceted. On the one hand, the expression of TNC is exacerbated following brain injuries such as stroke [51]. After experimental SAH, TNC is upregulated in the brain parenchyma [57]. The genetic ablation of TNC attenuates programmed neuronal cell death and neuroinflammatory responses, demonstrating TNC’s pathogenic contribution to early brain injury after SAH [57]. On the other hand, TNC may also exert beneficial effects on neuroplasticity. Studies suggest that TNC is involved in cellular mitotic activity and motility, phenotypic commitment, axonal pathfinding, myelin sheath formation, and synaptic adaptability [65]. Nevertheless, the impact of TNC on neuronal function appears to be ambivalent, and it is contingent upon the cell type, developmental stage, and environment [65].

As a glycoprotein of the ECM, TNC exhibits a complex role in neural plasticity promoting or inhibiting structural/functional reorganization, depending on the brain region, developmental stage, and pathological environment. In the neuropathic pain model of the spinal dorsal horn, TNC enhances the activity of astrocytes by activating the NR2B–NMDAR–JNK pathway, exacerbating pain transmission [54,130]. In the obesity model of the ARC region in the hypothalamus, the remodeling of PNNs results in the deposition of TNC. The negatively charged barrier of its CS-GAG chains hinders insulin from binding to agouti-related protein (AgRP) neurons, inducing insulin resistance [118]. In the adult hippocampus, TNC is upregulated with synaptic activity under normal physiological conditions. It interacts with integrin αvβ3 and TLR4, activates the ERK1/2 signaling pathway, and promotes dendritic spine formation and LTP. In contrast, in the chronic stress model, excessive TNC binds to the neural cell adhesion molecule (NCAM), inhibiting spine maturation by reducing the Ca^2+^ influx of NMDA receptors [73,106,107]. During the postnatal development stage, TNC deposition in the subplate/intermediate zone (SP/IZ) is dependent on the binding of neurocan (NCAN) to hyaluronic acid (HA). Specifically, NCAN interacts with TNC to form a ternary complex comprising HA, NCAN, and TNC. This complex is encountered by developing cortical neurons during their migration. Notably, the enzymatic or genetic disruption of the ternary complex delays radial migration via the inhibition of the multipolar-to-bipolar transition [144]. The enzymatic digestion of HA hinders the NCAN and TNC in the SP/IZ, which supports the function of HA as an ECM scaffold in the developing brain. Conversely, HA is capable of transducing extracellular signals to developing neurons through cell surface HA receptors, such as CD44 [145]. After cortical injuries such as stroke, TNC is cleaved by matrix metalloproteinase-9 (-9-9), releasing BDNF to promote axon sprouting [55,146]. TNC accumulates in layer V pyramidal neurons. By binding to BDNF with low affinity, it temporarily restricts BDNF-mediated axon growth, ensuring the proper pruning of neural circuits [107,146]. In the spinal cord, TNC is mainly expressed by reactive astrocytes. In the early stage of injury, it activates microglia through CD44, exacerbates inflammation, and inhibits axon regeneration [147,148,149]. However, in the chronic phase, TNC interacts with CSPGs to form a permissive scaffold, promoting axons to grow across the lesion site [148].

The balanced action of TNC in neuroinflammation and neuroplasticity may partly depend on its interactions with other ECM components. For instance, PNN is a highly cross-linked ECM, and it principally comprises by hyaluronan, proteoglycans, hyaluronan-binding proteins, and TNC [150]. PNN is predominantly found in GABAergic interneurons expressing the protein parvalbumin, where they have a crucial function in synaptic activity, cognitive processes (including learning and memory), oxidative stress regulation, and inflammatory responses [150]. The interaction between TNC and PNNs may modulate neuroinflammatory responses and influence synaptic plasticity. Moreover, the engagement of TNC with integrins may alter its role in neuroinflammation and neuroplasticity. TNC’s binding to αvβ1 and αvβ6 integrins can impact neuroinflammatory responses and synaptic plasticity, thereby orchestrating the overall influence of TNC.

Our study highlights TNC’s multifunctionality, which renders it a promising cross-disease target, while also presenting challenges related to specificity. TNC mediates neuroinflammation and regulates neuroplasticity, and it is involved in CNS injury repair, showing functional duality depending on the context. For instance, in Alzheimer’s, it worsens Aβ deposition and neuroinflammation but supports synaptic plasticity in a healthy hippocampus. In spinal cord injury, it initially inhibits axon regeneration but later aids repair. Thus, non-selective targeting could hinder beneficial roles while addressing pathological effects. TNC’s presence outside the CNS in areas such as hematopoietic organs, cardiovascular tissues, and tumors poses risks of off-target effects. CNS-targeted TNC interventions might unintentionally impact myocardial remodeling or glioblastoma progression, potentially worsening cardiovascular fibrosis or tumor metastasis. Additionally, TNC’s interactions with various molecules (e.g., TLR4, integrins, TGF-β) complicate targeting efforts, as inhibiting neuroinflammation could disrupt neuroplasticity and tissue homeostasis, upsetting the balance between inflammation control and neural repair. Future research should focus on creating context-specific targeting strategies to overcome specificity challenges. This includes designing ligands that selectively bind to disease-associated splice variants of TNC, such as fibronectin type III repeats 6–8 linked to hippocampal dysfunction in chronic stress, rather than its constitutive isoforms. Additionally, using tissue-specific delivery systems, such as CNS-penetrating nanoparticles, can restrict TNC modulation with respect to affected brain regions. Discussing these strategies strengthens this review by offering a balanced perspective on TNC’s therapeutic potential, highlighting both its benefits and the key challenges for clinical application.

## 8. Future Perspectives

Despite advancements in understanding the roles of TNC in neuroinflammation and neuroplasticity, significant gaps in knowledge persist. Future research should focus on several key areas: Firstly, the mechanisms underlying TNC’s functional duality should be elucidated. It is essential to clarify how factors such as cell type, developmental stage, and pathological conditions influence TNC’s transition between promoting or inhibiting plasticity and inflammation. This includes investigating TNC domain-receptor interactions, such as those between fibronectin III repeats and TLR4/integrins, in addition to species-specific variations in synaptogenesis. Additionally, examining TNC’s interactions with other extracellular matrix molecules, such as TNR and brevican, is crucial for understanding its role in maintaining the excitatory-inhibitory balance. Secondly, research should focus on mapping the remodeling of PNNs containing TNC. Employing high-resolution imaging and spatial transcriptomics can facilitate the tracking of TNC-PNN alterations in conditions such as AD (in the hippocampus), PD (in the substantia nigra), and PTSD (in the prefrontal cortex and amygdala). Developing context-specific interventions, such as the digestion of PNN chondroitin sulfate glycosaminoglycans (CS-GAGs), may help reverse neural rigidity and restore plasticity. Thirdly, it is essential to validate the clinical utility of TNC and its potential as a target for therapeutic interventions. This can be achieved by conducting studies involving large human cohorts to establish TNC as a biomarker for TBI and SAH by correlating serum and CSF levels with disease severity, the same can be achieved for AD via longitudinal assessments of its correlation with disease progression. Furthermore, therapeutic strategies should be refined to include the inhibition of the TNC-TLR4/MyD88/NF-κB signaling pathway to mitigate neuroinflammation, the utilization of TNC-derived fragments for stroke and spinal cord repair, and the targeting of splicing variants to minimize off-target effects. Fourthly, it is imperative to elucidate the mechanisms underlying the EV-mediated trafficking of TNC. This includes identifying the role of Cav1 in the sorting of TNC into specific EV subsets and investigating how EV-associated TNC influences glioblastoma pre-metastatic niches and neuroinflammatory foci. Additionally, engineering EVs for the targeted delivery of TNC inhibitors and neuroprotective fragments to facilitate their passage across the BBB is crucial. Collectively, these initiatives will enhance our understanding of TNC and expedite its translation into clinical interventions for CNS disorders.

## 9. Conclusions

TNC, a complex extracellular matrix protein within the CNS, plays a significant role in modulating both homeostatic and pathological processes. This review examines its involvement in neuroinflammation and neuroplasticity, in addition to its potential as a pharmacotherapeutic target. In the context of neuroinflammation, the upregulation of TNC is associated with the activation of microglia and astrocytes, resulting in the release of pro-inflammatory cytokines and subsequent neuronal damage. TNC interacts with TLR4 to enhance immune and inflammatory responses, contributing to the progression and cognitive decline in chronic conditions such as AD and PD. TNC is emerging as a promising biomarker correlating with the severity and prognosis of acute CNS injuries and as a therapeutic target. It facilitates neuroinflammation through the TLR4/MyD88/NF-κB signaling pathway, and inhibiting this pathway has been shown to reduce inflammation. Additionally, TNC regulates synaptic plasticity via interactions with integrins and PNN, with TNC deficiency shown to mitigate neuronal damage in preclinical models. In terms of neuroplasticity, TNC supports neural development by facilitating neuron and axon migration and synaptogenesis, although its aberrant overexpression can result in synaptic impairment. The ability of TNC to modulate inflammation and neural circuits positions it as a critical target for future research.

## Figures and Tables

**Table 1:** The effects of TNC in various CNS disorders on neuroinflammation vs. plasticity.

CNS Disorder	Neuroinflammation Effects	Plasticity Effects
Stroke	TNC ↑ → TNF-α/IL-1β release, BBB disruption, brain injury ↑ [17,51,52,53,54]	TNC ↓ → apoptosis ↓, tissue repair ↑ [55,56,57]
Traumatic Brain Injury (TBI)	CSF TNC ↑ → inflammation ↑ [58,59,60]	TNC ↑ → NSC migration ↑ [1,61] TNC ↑ → synaptic remodeling ↓ [51]
Alzheimer’s Disease (AD)	TNC ↑ → Aβ deposition ↑, inflammation ↑, cognitive decline ↑ [62,63]	TNC ↓ → Aβ ↓, synaptic function ↑, neuroprotection ↑ [62,64]
Parkinson’s Disease (PD)	TNC activates microglia via TLR4 → dopaminergic degeneration [29,65]	TNC ↓ → excitotoxicity ↓, PNN integrity disrupted → synaptic instability [29,66]
Epilepsy	TNC ↑ → seizure threshold ↓ [21,23]	TNC ↓ → PNN degradation ↑ → GABAergic inhibition ↓ → synaptic reorganization ↓ [23,66,67]

Note: The symbol “↑” represents the protein or related activity elevation, the symbol “↓” denotes the protein or related activity decrease, and the symbol “→” illustrates the progression to subsequent steps, implying “induces” or “triggers”. TNC: tenascin-C; TNF-α: tumor necrosis factor α; IL-1β: interleukin 1β; BBB: blood–brain barrier; CSF: cerebrospinal fluid; TLR4: toll-like receptor 4; NSC: neural stem cells; PNNs: perineuronal nets.

## Data Availability

Data available on request from the authors.

## Abbreviations

TNC: Tenascin-C; ECM: extracellular matrix; TLR4: Toll-like receptor 4; CNS: central nervous system; BDNF: brain-derived neurotrophic factor; AD: Alzheimer’s disease; PD: Parkinson’s disease; BBB: blood–brain barrier; LTP: long-term potentiation; LTD: long-term depression; FNIII repeats: fibronectin type III repeats; FGF: fibroblast growth factor; PDGF: platelet-derived growth factor; TGF-β: transforming growth factor-β; MyD88: myeloid differentiation factor 88; NF-κB: nuclear factor-κB; ICH: intracerebral hemorrhage; EVs: extracellular vehicles; Cav1: Caveolin-1; CSF: cerebrospinal fluid; SAH: subarachnoid hemorrhage; EBI: early brain injury; DCI: delayed cerebral ischemia; MAPK: mitogen-activated protein kinase; NMDA: N-Methyl-D-Aspartate; PNN: perineuronal nets; GABAergic: Gamma-Aminobutyric Acid-ergic; CSPGs: chondroitin sulfate proteoglycans; SSC: somatosensory cortex; PV: parvalbumin; CS-GAG: chondroitin sulfate–glycosaminoglycan; IL-1β: interleukin-1β; MD: monocular deprivation; LPS: lipopolysaccharide; ARC: arcuate nucleus; AgRP: agouti-related protein; NCAM: neural cell adhesion molecule; NCAN: neurocan; HA: hyaluronic acid.
